# Sandfly-Borne Phleboviruses in Portugal: Four and Still Counting

**DOI:** 10.3390/v14081768

**Published:** 2022-08-13

**Authors:** Fátima Amaro, Líbia Zé-Zé, Maria João Alves

**Affiliations:** 1 INSA—Centre for Vectors and Infectious Diseases Research, National Institute of Health Doutor Ricardo Jorge, Avenida da Liberdade n.-5, 2965-575 Águas de Moura, Portugal; 2 ISAMB—Instituto de Saúde Ambiental, Faculdade de Medicina da Universidade de Lisboa, Av. Prof. Egas Moniz, Ed. Egas Moniz, 1649-028 Lisboa, Portugal; 3 BioISI—Biosystems and Integrative Sciences Institute, Edificio TecLabs, Campus da FCUL, Campo Grande, 1749-016 Lisboa, Portugal

**Keywords:** phleboviruses, sandflies, Portugal

## Abstract

According to ICTV, there are currently 66 known phlebovirus species. More than 40 of these viruses were isolated or detected in phlebotomine sandflies and some of them are known pathogens. In Portugal, information about sandfly-borne phleboviruses is scarce and scattered sandfly-borne diseases are neglected and often not considered in differential diagnoses. The main objective of this work was to gather the existing information and to raise awareness about the circulating phleboviruses in this country. To date, Massilia and Alcube phleboviruses have been isolated from sandflies in southern Portugal. Human infections with Toscana and Sicilian phleboviruses have been reported, as well as seroprevalence in cats and dogs. More studies are needed in order to understand if the viruses isolated during the entomological surveys have an impact on human health and to fully understand the real importance of the already recognized pathogens in our country.

## 1. Introduction

The genus *Phlebovirus* is included in the *Phenuiviridae* family. According to ICTV, this genus currently comprises 66 viral species [1]. Phleboviruses, like all members of *Phenuiviridae*, are tri-segmented, with a negative-stranded RNA genome. In the case of phleboviruses, this genome includes a small segment (S) which encodes the nucleocapsid protein (N) and a smaller nonstructural protein (NS), a medium segment (M) responsible for encoding the non-structural protein (NSm) and two envelope glycoproteins (Gn and Gc) and finally, a large (L) segment which encodes the RNA-dependent RNA polymerase (RdRp) [2].

It is accepted that phlebovirus taxonomy is complex since it undertakes constant updates and rearrangements as new genomic data emerge due, not only to the high mutation rates because of the absence of error detection and correction activities by the viral polymerase, but also to the phenomenon of recombination. Presently, the phlebovirus species demarcation criteria determines that viruses with <95% identity in the amino acid sequence of RdRp, encoded by the L segment, are considered a unique species [3]. Nevertheless, reassortants may challenge this current definition of species, emphasizing the prospect that additional debate may be essential for determining when they may be considered as members of different species [3,4,5].

Nowadays, more than 40 phleboviruses species are known to have been isolated from or detected in phlebotomine sandflies (Diptera: Psychodidae, Phlebotominae) [5]. Phlebotomine sandflies can be found almost everywhere in the world and their distribution includes several countries with warm climate such as southern Europe, Asia, Africa, Australia and Central and South America [6]. In the subfamily, Phlebotominae, we can find the genera *Phlebotomus* and *Sergentomyia,* which are present in Eurasia and Africa, and the genus *Lutzomyia* dispersed through Central and South America. Sandflies in the genera *Phlebotomus* and *Lutzomyia* assume an important role in public health since they are recognized vectors, not only of pathogenic phleboviruses, but also (and mainly) *Leishmania* spp. Parasites which are responsible for an estimated 700,000 to 1 million new cases annually worldwide [7]. *Bartonella bacilliformis,* a bacterium which, to this day, is known to occur only in the Andes Mountains, in western South America, including Peru, Colombia, and Ecuador, is also transmitted by sandflies and responsible for Oroya fever and verruga Peruana (Peruvian warts) [8].

In Portugal, five species of sandflies are presently recognized: *Phlebotomus ariasi*, *Ph. Papatasi*, *Ph. Perniciosus*, *Ph. Sergenti* and *Sergentomyia minuta*. Phleboviruses have been isolated from all these five species in the Mediterranean region (e.g., *Toscana phlebovirus* from *Ph. perniciosus, Ph sergenti* and *S. minuta*; *Sicilian phlebovirus* from *Ph. ariasi and Ph papatasi*) [9,10]. Here we present a review of the sandfly-borne phleboviruses known to circulate in Portugal.

## 2. *Sicilian phlebovirus*

The *Sicilian phlebovirus* is a sandfly-borne virus only recently recognized as a species [11]. Nonetheless, the sandfly fever Sicilian virus (SFSV), belonging to the aforementioned species, was isolated by Albert Sabin in 1943, during World War II, from a serum sample collected from a soldier in the USA allied troops who fell ill after the Sicily landings, and again, in that same year during an outbreak of febrile disease in Egypt [12,13].

SFSV is mainly transmitted by *Ph. Papatasi* but other sandfly species, such as *Ph. Ariasi,* and other species of *Larroussius* group such as *Ph. Perniciosus*, *Ph. Neglectus* and *Ph. longicuspis,* may also transmit viruses from the *Sicilian phlebovirus* species [10,14,15].

After a 3–5-day incubation period, the SFSV infection is characterized by sudden and severe fever, accompanied by headaches, malaise, photophobia, myalgia and retro-orbital pain. This disease is often designated as pappataci fever, sandfly fever or three-day fever, since the febrile syndrome usually lasts two or three days [16]. In contrast with Toscana virus infections which can be neurotropic, SFSV infections in general are not believed to be associated with neurologic manifestations. Nevertheless, there is a report of a German 15-year-old girl who had been in Turkey, presenting severe meningitis after initial mild symptoms. According to the authors, ELISA and immunoblot confirmed infection with SFSV, and this was the first time SFSV was associated with neurological symptoms [17]. Another publication referring to central nervous system involvement of SFSV reported a 30-year-old man testing IgM positive in anti-SFV IgG and IgM detection immunoassays. Initially, the patient presented with acute gastroenteritis-like illness but, two days later, he developed severe encephalitis with status epilepticus [18]. In addition, there is a case, which remains as a probable infection of SFSV, where a patient who developed encephalitis with lethal consequences, was diagnosed through positive serum in ELISA IgM antibodies detection [19]. On the other hand, Ergunay and colleagues (2012) confirmed, through PCR and genome sequencing, a case of encephalitis in a 63-year-old woman infected with sandfly fever Turkey virus, a *Sicilian phlebovirus* species member [20]. Furthermore, and still referring to unusual manifestations, there is a report of skin lesions in two patients with PCR positive results for SFSV [21].

Human infections of SFSV or Sicilian-like phleboviruses have been reported in many countries of the Mediterranean region and the Middle East. Outbreaks or sporadic human cases have been described, for example, in Cyprus, Turkey, Iraq and Ethiopia [15,22,23,24,25,26,27,28,29,30]. In addition to those reported cases of acute disease, retrospective serological studies performed on humans presenting with febrile syndromes and/or compatible symptoms with SFSV infections, together with serosurveys of healthy individuals, indicate that *Sicilian phlebovirus*, or closely related viruses, circulate in three continents: Europe, Africa and Asia. In Europe, cases have been reported from countries such as France, Italy, Greece, Kosovo, Turkey and Cyprus [28,31,32,33,34,35,36,37,38,39]. In Africa, there are studies from Algeria, Sudan and Egypt [14,40,41], and in Asia, there are seroprevalence reports from Iran, Israel, Pakistan, Bangladesh and Afghanistan [42,43,44,45,46,47].

In 1976, Tesh and colleagues reported a very extensive study developed in 59 localities in Africa, the Mediterranean region, eastern Europe and Asia using neutralizing antibodies against eight phleboviruses and found out that SFSV circulated, among others, also in Croatia, Morocco, Somalia, Saudi Arabia, Moldova, Azerbaijan, Uzbekistan and Turkmenistan [48].

Regarding the circulation of *Sicilian phlebovirus* among vertebrates other than human, including wild and domestic mammals, several reports confirm the detection in different mammal species, including rodents, insectivores and carnivores in Tunisia, Morocco, Spain and Italy [49,50,51,52], bats in Spain [16], dogs in Greece, Cyprus and Tunisia [53,54] and livestock in Kosovo [55].

In Portugal, the first reference to the SFSV was made in 1974, when a survey for antibodies to arboviruses in human sera was carried out and hemagglutination inhibition assays showed four positive reactions (in 1690 tested samples) with the SFSV virus antigen [56]. Since then, no serological surveys have been performed and no human infections of SFSV were reported until the summer of 2017, when an eight-year-old boy presenting with fever, anorexia and mild headaches tested positive for antibodies against SFSV and then seroconverted [57,58]. This was the first time that SFSV infection was linked to a symptomatic infection in this country. The presence of the virus in Portugal is corroborated by several seroprevalence studies which found antibodies in 4.3% (17/400) of the studied human population in Setúbal district, southwest of Portugal, and in domestic animals such as cats (2.2%, 8/367) and dogs (50.8%, 581/1160; 56.3%, 327/581) [59,60,61,62].

## 3. *Toscana phlebovirus*

*Toscana phlebovirus* (TOSV) was isolated for the first time from *Ph. perniciosus* sandflies, collected in 1971 in Monte Argentario, Tuscany region, during an arbovirus study in Italy. Later, between 1980 and 1985, the virus was also isolated from *Ph. perfiliewi* in a study carried out in the same region whose objective was to determine the possible vectors and foci of TOSV and its importance with regard to human health [63].

It was only in the summer of 1983 that this virus was isolated for the first time from the cerebrospinal fluid (CSF) of a young woman with aseptic meningitis attending a hospital in the Tuscany region [64]. As such, it has now been recognized for a long time that TOSV is accountable, not only for asymptomatic or mild infections, but is also commonly associated with neurological disease, making it the most important sandfly-borne phlebovirus in terms of public health, and apparently, together with enteroviruses and herpesviruses, one of the three major viral pathogens responsible for aseptic meningitis reported during the warmer season in countries around the Mediterranean Basin [65,66].

A recent study estimated the incubation period of TOSV at 12 days [67]. Viremia persists only for two or three days, and cases can be diagnosed by direct isolation of the virus or RNA detection through reverse transcription PCR in CSF or blood [68]. In addition, there are records of TOSV RNA detection in urine samples and, more recently, of infectious TOSV in human semen which may indicate a potential for sexual transmission [69,70]. However, since the viremic period is short, the diagnosis of TOSV can be confirmed serologically, for example, through a four-fold or greater change in virus-specific quantitative antibody titers in paired sera or virus-specific IgM antibodies in serum with confirmatory virus-specific neutralizing antibodies in the same or a later specimen [71].

The most common clinical manifestations due to TOSV infections are fever, headache, nausea and vomiting, fatigue, photophobia, myalgia, febrile episodes, rash and stiff neck. Most infections, including the ones that lead to meningitis, encephalitis or meningoencephalitis, have a benign course and are self-limited; however, six fatal outcomes in elderly patients have been recorded: one in Italy and five in Romania [72,73]. Among the atypical clinical manifestations, with neurological involvement, reports of Guillain-Barré syndrome, hydrocephalus, hearing loss, speech problems, paresis, myositis and fasciitis, facial paralysis and personality changes can be found [74,75,76,77,78,79,80,81,82]. Testicular manifestations are also documented [76,83,84].

Until recently, TOSV was included in the *sandfly fever Naples virus* species but it is now considered a species of its own [11]. Furthermore, genetic studies have described the existence of diverse TOSV genotypes. In the latest review about TOSV, three lineages were confirmed to circulate in the Mediterranean area: lineage A strains in Italy, France, Turkey, Tunisia and Algeria; lineage B strains in Portugal, Spain, France, Morocco, Croatia and Turkey and lineage C in Croatia and Greece [85]. Co-circulation of strains A and B has been documented in France and Turkey and of strains B and C in Croatia. TOSV is also present in other Balkan countries such as Kosovo, Bosnia Herzegovina and Bulgaria, and in the Mediterranean islands of Elba, Baleares, Malta, Corsica, Sardinia, Cyprus and Crete [85]. Human seroprevalence of TOSV is usually around 10−24% but can reach 40% or higher within the endemic regions, as in the case of northern Tunisia where a microneutralization-based study detected a seroprevalence of 41% (522/1273) [86,87]. A seroprevalence as high 77.2% (278/360) was detected, for example, in sera samples collected in a high-risk population in Italy (Tuscany), and although the numbers varied significantly in the different regions, 37.5% (755/2016) of a studied resident population in Croatia presented IgM antibodies [88,89].

The natural cycle of TOSV remains unclear. Nevertheless, experimental infections of sandflies with the virus confirmed that both transovarian and venereal transmission may occur [90,91,92]. In addition, TOSV has also been isolated in nature from male and female sandflies [93]. The main vector is believed to be the sandfly *Ph. Perniciosus,* but whether there is a vertebrate reservoir is yet to be confirmed. The first isolation of TOSV from a non-human vertebrate was from a brain sample of a bat (*Pipistrellus kuhlii*) [63]. Additionally, TOSV sequences were identified in birds’ organs during the screening of avian specimens collected in the Mediterranean coast of the Anatolian peninsula, Turkey [94]. Viral isolation was not achieved but brain and kidney tissues from a greater flamingo (*Phoenicopterus roseus*), a great white pelican (*Pelecanus onocrotalus*) and a black stork (*Ciconia nigra*) tested positive for TOSV genotypes A and B in PCR assays [94]. TOSV has also been detected in other vertebrates, for example, in Granada (Spain), 48.3% (138/286) of dogs were seropositive for TOSV; seropositivity of 3.9% (9/231) was found in dogs on the eastern coast of Corsica and out of a total of 93 dogs, 4 were seropositive (4.3%) for TOSV in Kabylia [95,96,97].

To date, despite extensive efforts, TOSV has never been detected in entomological surveys conducted in wild-caught sandflies in Portugal and the first reference to the presence of this virus in the country was made in 1985 when its isolation from a tourist who became infected in Albufeira, in 1983, was reported in Sweden [98]. Therefore, Portugal was the second country, after Italy, to be considered endemic for TOSV [98]. Years later, in 1995, another tourist returned symptomatic to his country of origin, Germany, after being infected with TOSV in the region of Coimbra [99]. Subsequently, between 2002 and 2005, six more cases of TOSV were diagnosed, using molecular methods, in Portuguese patients with meningitis attending hospitals in the metropolitan area of Oporto, in the north of Portugal, but no genomic sequences were reported [100]. In a follow-up study, a seroprevalence of 3.9% (IgG) was found in 334 sera samples randomly collected from individuals who sought care in the hospitals in the same region [101].

In 2011, a report of a serological study performed at the National Reference Laboratory for vector-borne viruses (Centre for Vectors and Infectious Diseases Research, National Institute of Health, CEVDI/INSA) was published. This study included samples collected from 538 patients from all over the country, with and without neurological signs between 2004 and 2008 [102]. In house indirect immunofluorescence assay and commercial enzyme-linked immunosorbent assays were used. A prevalence of 4.2% (7/165) for IgG antibodies was found in the group of patients with neurological signs. Additionally, 3% (5/165) of the patients had IgG and IgM, revealing recent infections. In the group with no neurological signs, the IgG prevalence was 1.3% (5/373). In this study, only two patient samples were also confirmed with plaque reduction neutralization tests with the TOSV ISS. Phl.3 Italian strain, implying that different genotypes of TOSV virus may be circulating in Portugal.

In 2021, an update of TOSV cases in a total of 608 patients, whose samples were sent for laboratorial diagnosis at CEVDI/INSA between January 2008 and December 2018, referred to five acute TOSV infections. Three other patients presented serological evidence of previous contact with the virus [58]. Another recently published regional study (2022) involving only individuals from the Setúbal county, disclosed that out of 400 sera tested, 21 (5.3%) were positive for TOSV IgG [59].

Concerning seroprevalence studies in non-human vertebrates in Portugal, an investigation using in-house immunofluorescence assays performed in sera samples of wolves *(Canis lupus signatus*) and foxes (*Vulpes vulpes*) found that 1 in 49 wolves (2%) and 1 in 37 foxes (2.7%) presented IgG antibodies [103]. In another report using the same technique, 100 healthy military dogs were screened and one tested IgG positive for TOSV [104]. Other studies involving neutralization assays in pets detected seroprevalence for TOSV: 6.8%, (79/1160) and 6.2% (36/581) in dogs and 3.7% (7/189) and 4.9% (18/365) in cats. [60,61,62].

## 4. *Massilia phlebovirus*

*Massilia phlebovirus* was isolated for the first time from pools of *Ph. perniciosus* sandflies collected in the suburban area of Marseille, France, in July 2005 [105]. In order to assess the possible existence of human infections, a retrospective study aiming at the detection of *Massilia phlebovirus* RNA was performed in 477 CSF samples from local patients presenting central nervous system disease between 2002 and 2006. The results were all negative [105].

Sanchéz-Seco and colleagues, in 2010, reported the detection of Massilia-like virus in sandflies collected in Catalonia, Spain [106]. In the same country, during a survey in sandflies in Granada province, a phlebovirus was isolated, fully sequenced and tentatively named Granada virus (GRV) [107]. GRV was, by that time, described as a new phlebovirus, likely to be a natural reassortant of the Massilia virus [107]. Antibodies against GRV were investigated in sera from healthy individuals collected in the Granada province, in 2003. From a total of 248, 37 (14.9%) tested positive by immunofluorescence assay. To discard cross-reactivity with TOSV, the positive sera were tested for neutralizing antibodies against GRV, and five samples retrieved positive results [107].

In a subsequent study conducted in the same region, IgG detection through immunofluorescence assays was carried out in asymptomatic individuals and IgM detection was performed (with the same method) in patients attending healthcare facilities during the warmer months [108]. In total, 145 healthy individuals out of 920 (15.8%) were positive for anti-GRV antibodies, and from these, 17.9% (*n* = 26) were positive for anti-GRV neutralizing antibodies. In the group of symptomatic individuals, 36 out of 547 were IgM positive. The authors of the study concluded that GRV may infect humans in that region and, as expected, most cases would probably cause no symptoms [108].

Despite being initially regarded as a new virus, considering the new criteria for species demarcation, GRV seems, in fact, to belong to *Massilia phlebovirus* species [4]. The fact that human seroprevalence was found for GRV may suggest that *Massilia phlebovirus* can infect humans. However, it is not possible to assume that this species can cause human disease.

During a phlebotomine survey conducted in the south of Portugal during the summer in 2007 and 2008, a phlebovirus, tentatively named Arrábida virus, was isolated from sandflies collected in Arrábida region, Setúbal county. After whole genome sequencing, as in the case of GRV, it was believed that we were in the presence of a new reassortant phlebovirus [109]. However, once again, following the ICTV criteria, Arrábida virus must be considered a member of *Massilia phlebovirus*. In the same survey it was possible to detect and fully sequence two other *Massilia phlebovirus* variants (Massilia virus isolate 127 in Arrábida region and Massilia virus isolate 130 in eastern Algarve, Olhão county) and to isolate and fully sequence two other Massilia variants in sandflies collected, once again, in Arrábida: PoSFPhlebV/21/2007 and PoSFPhlebV/70/2007 [4,110].

In agreement with the gathered data, *Massilia phlebovirus* is known to circulate in Portugal, Spain and France. To date, no seroprevalence studies have been carried out in Portugal.

## 5. *Alcube phlebovirus*

*Alcube phlebovirus* was isolated from a pool of sandflies collected in Arrábida region, in July 2007 [110]. It was firstly described as a new phlebovirus species clustering with members of the Salehabad virus species complex and forming a distinct monophyletic lineage with Arbia, (isolated from sandflies in Italy), Salehabad (first isolated from sandflies in Iran) and Adana (isolated from sandflies in Turkey) viruses [64,109,111,112]. According to the new taxonomic arrangement [1], all these viruses are currently recognized as different species, except for Arbia virus which is currently included in *Medjerda phlebovirus* species [5].

In general, the species formerly included in the Salehabad group were not considered to be of significant medical or veterinary interest [113]. Nevertheless, high seroprevalence rates of *Adana phlebovirus* have been found in animals such as goats, sheep and dogs, and low seroprevalence rates have been identified in the human population [112]. On the other hand, genomic RNA of Adria virus (belonging to *Salehabad phlebovirus* species) was first detected in sandflies in Albania, and later in Greece in a child with febrile syndrome and convulsions, which may suggest its pathogenicity [114,115]. Further studies are needed in order to clarify whether *Alcube phlebovirus* infects humans.

## 6. Other Genome Detections of Phleboviruses in Sandfly Pools in Portugal

During the entomological surveys performed across two regions in the south of Portugal in the summers of 2007, 2008 and 2018, other RNA detections, unpublished until now, were achieved in 22 pools of sandflies in addition to the already fully sequenced and mentioned *Alcube* and *Massilia phleboviruses* strains.

For the additional detections, isolation attempts, performed as previously described [110], were not successful after at least three blind passages in Vero E6 cells, probably due to sample degradation. Thus, further investigation was not feasible. Nevertheless, it was possible to obtain partial sequences for the S segment of 13 phleboviruses strains [116,117], four for the M segment (this study) and five for the L segment [117] in this study. For the segment M sequence amplification, primers PhlebMF1 (5′-CTCKATTGAAKATKGCCATKGG-3′) and PhleboMR1 (5′-ATGCTTTGAGCAGAGYGGWGG-3′) were specifically designed to amplify a 494 bp partial sequence of viruses related to MASV, GRAV and TOSV. For pool 149/2008, amplification of a 1234 bp partial sequence of RdRp (segment L) was obtained using primers MASV_LF2 (5′-CTGACAAGGCTGACGGTTCT-3′) and MASV_LR2 (5′-TGTACCAACGCCACGATTGA-3′) designed to amplify Massilia virus genome. Briefly, for the primers designed in this study, 5 μL of RNA and 10 pmol of each primer were added to SuperScript^®^ One-Step RT-PCR with Platinum^®^ Taq (Invitrogen by Life Technologies, Carlsbad, CA, USA). Polymerase chain reaction (PCR) conditions were as follows: reverse transcription at 50 °C for 30 min, denaturation at 95 °C for 5 min, 45 cycles of 94 °C for 20 s, 55 °C for 60 s and 72 °C for 60 s, and a final extension at 72 °C for 5 min. To increase sensitivity, a second round PCR was performed using the same primers and high fidelity PCR master (Roche, Mannheim, Germany). The obtained amplicons were purified after gel agarose visualization, using JETquick PCR Product Purification Spin kit (GENOMED GmbH, Löhne, Germany) and sequenced bi-directionally using ABI Prism 3130 Genetic Analyzer (Applied Biosystems, Foster City, CA, USA). Homology searches within the GenBank data set were performed using the BLASTn algorithm [118]. GenBank accession numbers of all new sequences reported in this study are presented in Table 1. Phleboviruses partial sequences were aligned with sequences available from GenBank using Clustal W within BioEdit version 7.2.5 [119] and manually edited whenever necessary (alignments are available in Supplementary Material). Phylogenetic analysis was performed using partial nucleotide sequences of nucleocapsid (*n* = 33), glycoprotein precursor (*n* = 20) and RNA-dependent RNA polymerase genes (*n* = 22 and *n* = 19). Maximum likelihood phylogenetic trees were estimated in Mega version X software [120] by using the best-fit model of nucleotide substitution as indicated by the Best DNA/Protein Model application (implemented in Mega X) [121]. The robustness of the inferred tree was tested by 1000 bootstrap replications.

**Figure 1:**
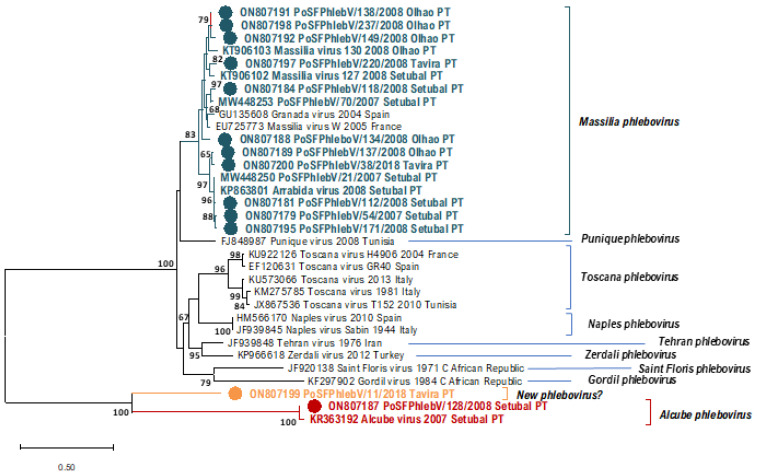
Maximum likelihood phylogenetic tree of phleboviruses’ partial nucleocapsid nucleotide sequences (segment S, 674 positions in the final dataset) using Kimura 2-parameter model and discrete gamma distribution. Sequence names in blue (*Massilia phlebovirus*), in red (*Alcube phlebovirus*), and yellow (New phlebovirus?) were detected in Portugal (PT). Sequence names marked with a colored bullet have been sequenced in this study. Bootstrap support over 60% is presented on branches. Used sequence alignment is available in Supplementary File S1: Phleboviruses’ nucleotide partial sequence alignment of S segments. Names of the sequences in the tree match the IDs in the alignment made available as a Supplementary File.

**Figure 2:**
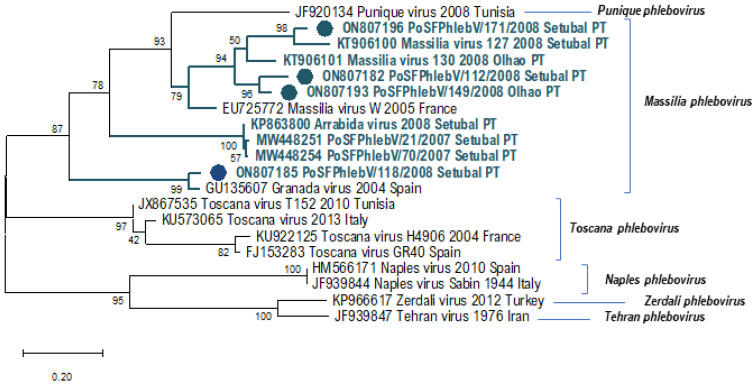
Maximum likelihood phylogenetic tree of phleboviruses partial glycoprotein precursor nucleotide sequences (segment M, 461 positions in the final dataset) using Tamura 3-parameter model and discrete gamma distribution. Sequence names in blue (*Massilia phlebovirus*) were detected in Portugal (PT). Sequence names marked with a colored bullet have been sequenced in this study. Bootstrap support over 60% is presented on branches. Used sequence alignment is available in Supplementary File S2: Phleboviruses’ nucleotide partial sequence alignment of M segments. Names of the sequences in the tree match the IDs in the alignment made available as a Supplementary File.

**Figure 3:**
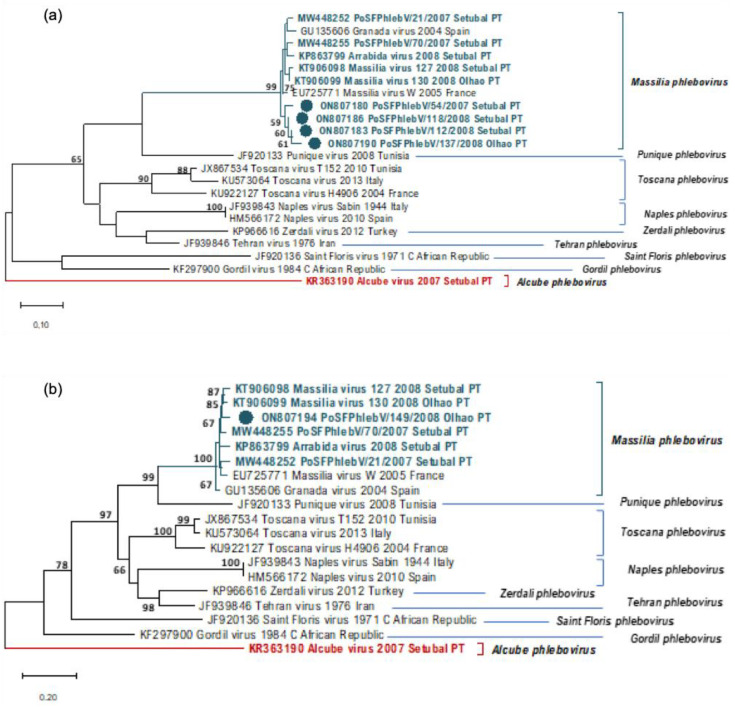
Maximum likelihood phylogenetic trees of phleboviruses partial RNA-dependent RNA polymerase nucleotide sequences (segment L). Sequence names in blue (*Massilia phlebovirus*) and in red (*Alcube phlebovirus*) were detected in Portugal (PT). Sequence names marked with a colored bullet have been sequenced in this study. Bootstrap support over 60% is presented on branches. Names of the sequences in the tree match the IDs in the alignment made available as a Supplementary File. (**a**) Maximum likelihood phylogenetic tree of phleboviruses partial RNA-dependent RNA polymerase nucleotide sequences (segment L, 227 positions in the final dataset) using Tamura 3-parameter model and discrete gamma distribution. Used sequence alignment is available in Supplementary File S3: (**a**) Phleboviruses’ nucleotide partial sequence alignment of L segments. (**b**) Maximum likelihood phylogenetic tree of phleboviruses partial RNA-dependent RNA polymerase nucleotide sequences (segment L, 1065 positions in the final dataset) using general time reversible model and discrete gamma distribution. Used sequence alignment is available in Supplementary File S4: (**b**) Phleboviruses’ nucleotide partial sequence alignment of L segments.

In Table 1, we can see the sequence data of all the phleboviruses detected/isolated from sandfly pools so far in Portugal. All the additionally detected phlebovirus sequences show a close relation to *Massilia phlebovirus,* except for PoSFPhlebV/128/2008 presumptively identified as *Alcube phlebovirus* through N gene partial sequence similarity (Table 1 and Figure 1, Figure 2 and Figure 3). The available data do not support a closer similarity of variants related to their geographic location detection (Setúbal *versus* Algarve region).

In the absence of complete genome sequences, it is not possible to draw firm conclusions about the genetic diversity of all detected viruses. Nevertheless, the similarity results obtained from BLASTn analysis (data not shown) and the branch changes in the trees for each genome segment (Figure 1, Figure 2 and Figure 3) indicate that some of these *Massilia phlebovirus* related viruses may represent reassortants. For instance, from the segment S partial sequence similarity analysis for PoSFPhleb/118/2008, it seems that this variant is closely related to *Massilia phlebovirus* PoSFPhleboV/70/2007 (Figure 1). However, using the segment M partial sequence, a closer similarity is observed with the GRV virus sequence (Figure 2). On the other hand, analyzing segment L partial sequences (Figure 3), no indication of a closely related ancestor of PoSFPhleb/118/2008 and the former *Massilia phlebovirus* variants is observed.

Branch changes are also clearly observed for PoSFPhlebV/112/2008 when analyzing different genome segments available sequences showing closer proximity to *Massilia phlebovirus*: Arrábida virus and PoSFPhleb/21/2007 in segment S, Massilia virus 127 and 130 in segment M and none of them in segment L sequences.

The partial gene N sequence detected in pool 11/2018 (PoSFPhlebV/11/2018; GenBank accession ON807199) shows a higher distance from all the other phleboviruses’ sequences. Although the available data are insufficient to enable the species identification, it seems to indicate potential circulation, in the Algarve region, of a new phlebovirus more closely related to *Alcube Phlebovirus*.

## 7. Discussion

In the last decades, entomological and virological studies have been expanding [122]. Concomitantly, the advances in laboratory techniques, particularly in molecular diagnostics, have enabled great strides in the identification and characterization of novel phleboviruses, resulting in a considerable increase in the number of recognized members of this genus. Phleboviruses may be responsible for more human diseases than previously thought [123]. However, as most native adults in endemic regions are immune, there are relatively few reports of clinical cases among indigenous people, in addition to the fact that mild symptoms do not frequently lead people to seek health care [23]. Often, reported cases of more severe infections are from travelers visiting endemic regions [124,125,126,127]. Yet, and surprisingly, some clinicians are still not considering TOSV and phleboviruses, in general, as causative agents of disease. Reports of the expansion of vector sandflies to more central countries in Europe, such as Germany, Switzerland and Austria, indicate that sandflies are expanding toward the north and raise awareness for the increasing risk for sandfly-borne diseases in territories not previously considered [128,129,130,131].

The first reference of a phlebovirus in Portugal was made in 1974, regarding human seroprevalence of *Sicilian phlebovirus*. This phlebovirus remained unnoticed in the human population until 2017. This species is distributed in three continents, and outbreaks caused by its members are known to occur from time to time in different parts of Eurasia and Africa. *Cyprus phlebovirus* (Sicilian-like) was recently indicated as the cause of a life-threatening condition in a 3.8-year-old child in Italy. It was the first time this variant was detected outside Cyprus [132]. This should be a warning sign that not only national reference centers, but also hospital microbiology laboratories should be equipped for systematic phleboviruses testing of patients presenting with febrile illness and central and peripheral nervous system febrile manifestations [53].

TOSV, which is also a widespread virus affecting two continents, causes infections leading to mild or neurological disease from north to south of Portugal. The fact that the only complete genomic sequences displayed in GenBank are from the S segment of the virus isolated in 1983 from a Swedish tourist corroborates that this virus is not being given its due importance in our country. For example, in Spain, in an update of TOSV neurological infection in Andalusia, from 1988 to 2020, TOSV was the second agent detected in CSF samples between April and November. In Granada province, considered as an hyperendemic area for TOSV, a seroprevalence of 25% was found [133]. Moreover, Collao and colleagues (2009) reported that sequences independently obtained in two laboratories from strains allegedly obtained from the same patient, infected in Portugal, clustered in different clades, genotypes A and B [134,135,136]. Indisputably, investigations of infections in the central nervous system and fever of unknown origin are needed in order to better understand the epidemiology of TOSV in Portugal and which genotypes of this virus are circulating. Also, other entomological investigations are necessary in Portugal.

The lack of genomic detection of phleboviruses in cats and dogs in Portugal is in line with studies from other regions. For instance, in an experimental infection of dogs with TOSV and *Sicilian phlebovirus* in Spain, it was demonstrated that healthy domestic dogs do not exhibit susceptibility to infection by these two viruses. The subjects did not show the development of noteworthy viremia nor expelled the viruses after being experimentally infected. Based on these results, dogs do not seem to be natural reservoir hosts of infection, nor play a meaningful part in phleboviruses’ transmission cycles [137]. As for wild mammals, a seroprevalence study of TOSV and Sicilian phleboviruses performed in bat colonies from southern Spain showed that, despite positive sera for both viruses found in different species, it is unlikely that these mammals play an important role in the biological cycle of the viruses in question [16].

MASV, with unknown pathogenic potential, was detected in entomological surveys in Setúbal and Faro, two districts in the south of Portugal. This fact leads us to believe that the virus may be widely spread in the country, or, at least, all over the south since the locations where this species was found are not contiguous. Even though complete genome sequences were not possible to achieve for the newly presented phleboviruses, analysis of their partial sequences, along with the complete genomes available within *Massilia phebovirus* species, highlights an impressive sequence diversity and potential for recombination/reassortment, which may explain the difficulty in detecting phleboviruses by PCR in surveillance studies. Further entomological surveys would be necessary to clarify this assumption, and human seroprevalence investigations could help to clarify if this virus is infectious to humans.

To date, *Alcube phlebovirus* has been detected only in Setúbal county, south of Portugal. Further investigation is needed to clarify its distribution and importance to public health which remains, as yet, undetermined.

## 8. Conclusions

Four phleboviruses are known to circulate in Portugal: *Sicilian phlebovirus*, *Toscana phlebovirus*, *Alcube phlebovirus* and *Massilia phlebovirus*. The first two are known pathogens in the Mediterranean Basin and responsible for cases of febrile illness or neurological disease in the summer months. The other two have not yet been classified as pathogens but further epidemiological studies must be performed in order to clarify this matter.

The possibility that a wider number of unidentified phleboviruses is circulating in Portugal should be considered. Furthermore, the fact that Alcube and Massilia viruses were isolated in 2007, at the very same location, meaning that co-circulating viruses can be found, raises concerns, especially due to the genomic characteristics of these viruses. Multiple infections in arthropods may favor reassortments of the genome segments and the major concern is that this process may modify the phleboviruses’ biological properties or originate viruses with unknown pathogen capacity. As such, the characterization of novel members of this genus and the competency to acknowledge the existence of reassortants is of utmost importance to prevent the occurrence of outbreaks of these emerging pathogens.

## Figures and Tables

**Table 1:** Phleboviruses detected and/or isolated from sandfly pools in Portugal.

Designation in GenBank	Collection Date	Collection County	S Segment (nr of bp/)	M Segment (nr of bp)	L Segment (nr of bp)	Accession Number	Species ID	Reference
Alcube virus strain S20 ^1^	July 2007	Setúbal	1758	4164	6405	KR363190-192	*Alcube phlebovirus*	[110]
PoSFPhlebV/21/2007 ^1^	July 2007	Setúbal	1854	4221	6404	MW448250-252	*Massilia phlebovirus*	[4]
PoSFPhlebV/54/2007 ^1^	August 2007	Setúbal	608	-	227	ON807179-180	Massilia phlebovirus *	This publication
PoSFPhlebV/70/2007 ^2^	September 2007	Setúbal	1873	4229	6386	MW448253-255	*Massilia phlebovirus*	[4]
PoSFPhlebV/112/2008 ^2^	May 2008	Setúbal	547	461	224	ON807181-183	Massilia phlebovirus *	This publication
PoSFPhlebV/118/2008 ^2^	June 2008	Setúbal	670	461	227	ON807184	Massilia phlebovirus *	This publication
Arrabida virus strain PoSFPhlebV/126/2008 ^1^	June 2008	Setúbal	1840	4198	6391	KP863799-801	*Massilia phlebovirus*	[109]
Massilia virus strain 127 ^1^	June 2008	Setúbal	1864	4225	6404	KT906098, 100, 102	*Massilia phlebovirus*	[110]
PoSFPhlebV/128/2008 ^1^	June 2008	Setúbal	310	-	-	ON807187	Alcube phlebovirus *	This publication
Massilia virus strain 130 ^3^	June 2008	Olhão	1864	4225	6404	KT906099, 101, 103	*Massilia phlebovirus*	[110]
PoSFPhlebV/134/2008 ^3^	June 2008	Olhão	338	-	-	ON807188	Massilia phlebovirus *	This publication
PoSFPhlebV/137/2008 ^3^	June 2008	Olhão	653	-	212	ON807189-190	Massilia phlebovirus *	This publication
PoSFPhlebV/138/2008 ^3^	June 2008	Olhão	670	-	-	ON807191	Massilia phlebovirus *	This publication
PoSFPhlebV/149/2008 ^4^	June 2008	Olhão	392	461	1041	ON807192-194	Massilia phlebovirus *	This publication
PoSFPhlebV/171/2008 ^5^	July 2008	Setúbal	333	461	-	ON807195-196	Massilia phlebovirus *	This publication
PoSFPhlebV/220/2008 ^6^	August 2008	Tavira	631	-	-	ON807197	Massilia phlebovirus *	This publication
PoSFPhlebV/237/2008 ^4^	September 2008	Olhão	452	-	-	ON807198	Massilia phlebovirus *	This publication
PoSFPhlebV/11/2018 ^7^	May 2018	Tavira	381	-	-	ON807199	New phlebovirus?	This publication
PoSFPhlebV/38/2018 ^7^	June 2018	Tavira	530	-	-	ON807200	Massilia phlebovirus *	This publication

^1^ Sheep pen in a farm; ^2^ hennery near a country house; ^3^ pigeonry near a country house; ^4^ hennery, near a country house; ^5^ kennel in a dog shelter; ^6^ hennery in a farm and ^7^ hennery near a country house. Strains with the same superscript number were collected at the same location. * Presumptive species ID based on available sequence analysis.

## Data Availability

Not applicable.

## Supplementary Materials

The following supporting information can be downloaded at: https://www.mdpi.com/article/10.3390/v14081768/s1, File S1: Phleboviruses’ nucleotide partial sequence alignment of S segments, File S2: Phleboviruses’ nucleotide partial sequence alignment of M segments, File S3: (a) Phleboviruses’ nucleotide partial sequence alignment of L segments, File S4: (b) Phleboviruses’ nucleotide partial sequence alignment of L segments.
